# Molecular Mechanisms of Acute Oxygen Sensing by Arterial Chemoreceptor Cells. Role of Hif2α

**DOI:** 10.3389/fphys.2020.614893

**Published:** 2020-11-23

**Authors:** Patricia Ortega-Sáenz, Alejandro Moreno-Domínguez, Lin Gao, José López-Barneo

**Affiliations:** ^1^Instituto de Biomedicina de Sevilla (IBiS), Hospital Universitario Virgen del Rocío/CSIC/Universidad de Sevilla, Seville, Spain; ^2^Departamento de Fisiología Médica y Biofísica, Facultad de Medicina, Universidad de Sevilla, Seville, Spain; ^3^Centro de Investigación Biomédica en Red sobre Enfermedades Neurodegenerativas (CIBERNED), Madrid, Spain

**Keywords:** carotid body, glomus cells, acute O2 sensing, electron transport chain, mitochondrial signaling, ion channels, mechanism of disease, paraganglioma

## Abstract

Carotid body glomus cells are multimodal arterial chemoreceptors able to sense and integrate changes in several physical and chemical parameters in the blood. These cells are also essential for O_2_ homeostasis. Glomus cells are prototypical peripheral O_2_ sensors necessary to detect hypoxemia and to elicit rapid compensatory responses (hyperventilation and sympathetic activation). The mechanisms underlying acute O_2_ sensing by glomus cells have been elusive. Using a combination of mouse genetics and single-cell optical and electrophysiological techniques, it has recently been shown that activation of glomus cells by hypoxia relies on the generation of mitochondrial signals (NADH and reactive oxygen species), which modulate membrane ion channels to induce depolarization, Ca^2+^ influx, and transmitter release. The special sensitivity of glomus cell mitochondria to changes in O_2_ tension is due to Hif2α-dependent expression of several atypical mitochondrial subunits, which are responsible for an accelerated oxidative metabolism and the strict dependence of mitochondrial complex IV activity on O_2_ availability. A mitochondrial-to-membrane signaling model of acute O_2_ sensing has been proposed, which explains existing data and provides a solid foundation for future experimental tests. This model has also unraveled new molecular targets for pharmacological modulation of carotid body activity potentially relevant in the treatment of highly prevalent medical conditions.

## Introduction

Oxygen (O_2_) is essential for survival of mammalian cells due to its role in numerous biochemical reactions, in particular, in mitochondrial ATP synthesis by oxidative phosphorylation. O_2_ deficiency (hypoxia), even if transient, can produce irreversible cellular damage. Chronic and acute adaptive responses to hypoxia have evolved to favor O_2_ homeostasis. Sustained (chronic) hypoxia, lasting hours or days, induces a powerful and generalized transcriptional response characterized by the expression of a broad cohort of genes that, among other changes, favors glycolysis, to obtain non-aerobically ATP, as well as angiogenesis and increased red blood cell number to enhance the O_2_-carrying capacity of the blood and its distribution to the tissues. Modulation of O_2_-sensitive genes depends on a family of prolyl hydroxylases (PHD), which use O_2_ as a substrate to hydroxylate and regulate the activity of hypoxia inducible transcription factors (HIFs; see for a recent comment Lopez-Barneo and Simon, 2020). To date, the PHD-HIF signaling pathway has been reported to modulate over 2,000 transcripts, many of them critically involved in numerous pathophysiological processes such as embryogenesis, stem cell fate and differentiation, tissue regeneration, inflammation and cancer, among others (Ratcliffe, 2013; Semenza, 2014; Colgan et al., 2020).

**Figure 1:**
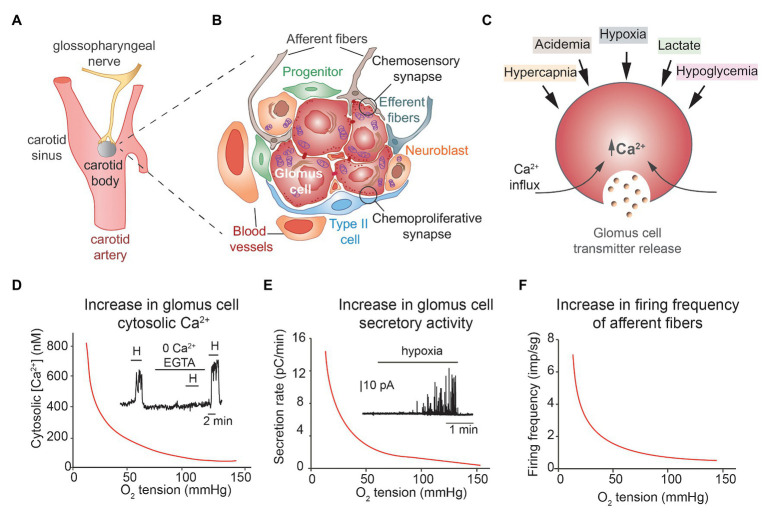
Structural and functional properties of carotid arterial chemoreceptors. **(A)** Location and innervation of the carotid body (CB). **(B)** Schematic representation of a CB glomerulus with indication of the various structural components and cell types. **(C)** Schematic representation of CB glomus cell activation by several stimuli. **(D,E)** Hyperbolic relationships between cytosolic Ca^2+^ (inset in **D**) and catecholamine release (inset in **E**) in single glomus cells as a function of oxygen tension in the external solution. Hypoxia-induced increase in cytosolic Ca^2+^ depends on extracellular Ca^2+^ influx **(D)**. **(F)** Relationship between firing frequency in afferent fibers of the sinus nerve as a function of blood oxygen tension. **(A–C)** Modified from Ortega-Saenz and Lopez-Barneo (2020). **(D,E)** Modified from Montoro et al. (1996) and Ortega-Saenz et al. (2006). **(F)** Modified from Lopez-Barneo (1996).

Exposure to hypoxia, as it occurs in high altitude or in patients with altered gas exchange in the lungs, also induces acute adaptive responses (hyperventilation and sympathetic activation) that in few seconds increase O_2_ uptake and its distribution to tissues. These life-saving cardiorespiratory reflexes are mediated by specialized cells in the homeostatic acute O_2_-sensing system (Weir et al., 2005). The prototypical acute O_2_-sensing organ is the carotid body (CB), a small arterial chemoreceptor located in the carotid bifurcation, which contains chemosensory and neurosecretory glomus cells (Figure 1A). Glomus cells release transmitters during exposure to hypoxia and other stimuli to activate afferent fibers of the glossopharyngeal nerve terminating at the brainstem respiratory and autonomic centers. Although it is over 30 years that the basic cellular physiology of the CB was described (see for a recent review Ortega-Saenz and Lopez-Barneo, 2020), the molecular mechanism underlying acute O_2_-sensing by glomus cells has remained elusive. Among the several attractive hypotheses postulated are the involvement of a specific NADPH oxidase, activation of AMP kinase during hypoxia, the reversible fast regulation of ion channels by gasotransmitters such as carbon monoxide and hydrogen sulfide, or the expression of an atypical olfactory receptor (Olfr78; see for recent reviews Lopez-Barneo et al., 2016; Rakoczy and Wyatt, 2018). Although all these processes can influence glomus cell function, none of them seem to be essential for acute O_2_ sensing because the various mouse models generated after ablation of the genes coding the relevant enzymes or receptors showed CB with practically normal responsiveness to hypoxia (He et al., 2002; Ortega-Saenz et al., 2006; Mahmoud et al., 2016; Wang et al., 2017; Torres-Torrelo et al., 2018). It has recently been reported that Olfr78-deficient CB cells have decreased responsiveness to mild hypoxia (Peng et al., 2020). Olfr78 is one of the most abundant mRNA species expressed in CB glomus cells (Zhou et al., 2016; Gao et al., 2017), as other highly expressed G-protein-coupled receptors, which may influence the input/output properties of chemoreceptor cells (Nurse, 2014; Ortega-Saenz and Lopez-Barneo, 2020). In recent years new experimental data have provided strong support for a “mitochondrial-to-membrane signaling (MMS) model” of acute O_2_ sensing, which combines the “membrane” and “metabolic” hypotheses. Here, after a succinct presentation of the general properties of CB glomus cells, we focus on the description of the MMS model of acute O_2_ sensing. We also discuss the potential medical implications of recent advances in CB research.

## Properties of Polymodal Carotid Body Chemoreceptor Cells

The CB is organized in clusters of cells called glomeruli. Each glomerulus contains neuron-like and tyrosine hydroxylase (TH)-positive glomus (or type I) cells, which appear grouped (normally 4–8 units) in the center, enveloped by processes of glia-like, glial fibrillary acidic protein (GFAP)-positive, type II or sustentacular cells (Figure 1B). Glomus cells have large nuclei, abundant mitochondria, and numerous secretory vesicles, containing dopamine, ATP, acetylcholine, and several other neurotransmitters and neuropeptides. These cells establish chemical synapses with afferent fibers (“chemosensory synapses”; Figure 1B) originating in the petrosal ganglion. It is well-established that the main transmitter in the chemosensory synapse is ATP, which binds to postsynaptic P2X receptors, although acetylcholine may also have a stimulatory effect (Zhang et al., 2000; Rong et al., 2003; Shirahata et al., 2007). Dopamine has an auto or paracrine role inhibiting Ca^2+^ channels in glomus cells (Benot and Lopez-Barneo, 1990) and, in addition, it can also inhibit postsynaptic HCH cationic channels in petrosal afferent neuros *via* D2 receptors (Zhang et al., 2018). Although mature O_2_-sensitive glomus cells seem to be post-mitotic, the CB also contains a population of immature TH-positive cells, normally localized in the periphery of the glomerulus, with fewer secretory vesicles and smaller sensitivity to hypoxia than mature glomus cells (Sobrino et al., 2018). In hypoxic conditions, these TH-positive “neuroblasts” proliferate and differentiate into mature glomus cells and in this way contribute to adult CB growth, a plastic CB response that increases the stimulatory input to the respiratory center and thereby facilitates chronic adaptation to hypoxic environments. Glomus cells also establish numerous chemical synapses with type II cells (Platero-Luengo et al., 2014). Indeed, transmitters released from glomus cells can induce ATP release from type II cells to potentiate the chemosensory synapse (Xu et al., 2003; Zhang et al., 2012). GFAP-positive type II cells, or a subpopulation of them, are quiescent multipotent stem cells that upon exposure to hypoxia are activated to proliferate and differentiate into new glomus cells, endothelial cells, and smooth muscle (Pardal et al., 2007; Navarro-Guerrero et al., 2016; Annese et al., 2017). Glomus cells and type II cells form “chemoproliferative synapses” (Figure 1B), such that neurotransmitters and neuromodulators (endothelin-1 among others) released from glomus cells (Chen et al., 2002) induce type II cells to exit the quiescent state and to start proliferating and differentiating (Platero-Luengo et al., 2014). Therefore, the adult CB is a sophisticated germinal niche that contains differentiated cells with complex sensory functions, as well as immature neuroblasts and progenitors with strong neurogenic and angiogenic potential that support the structural plasticity of the organ.

Chemosensory glomus cells are small (~10–15 μm in diameter) and electrically compact elements able to generate action potentials repetitively due to the expression of voltage-gated Na^+^, Ca^2+^, and K^+^ channels. They also express a broad spectrum of other ion channels types, notably background K^+^ channels, in particular, TASK1 and TASK 3 channels, and cationic TRP channels (Zhou et al., 2016; Gao et al., 2017). It is established that hypoxia produces glomus cell depolarization due to the inhibition of background and voltage-gated K^+^ channels; this leads to the opening of voltage-dependent Ca^2+^ channels, extracellular Ca^2+^ influx, and exocytotic transmitter release (Lopez-Barneo et al., 1988; Buckler and Vaughan-Jones, 1994; Urena et al., 1994). It has also been reported that the rise in intracellular Ca^2+^ can activate Ca^2^-permeant background cation channels to further potentiate Ca^2+^ entry and transmitter release (Kang et al., 2014). In addition to hypoxia, glomus cells are activated by hypercapnia, low extracellular pH, low glucose, and lactate as well as by hypoperfusion and several circulating hormones and cytokines. Although these stimuli utilize separate transduction mechanisms, they all converge on extracellular Ca^2+^ influx and the generation of a cytosolic Ca^2+^ signal that triggers transmitter release (see for a recent review Ortega-Saenz and Lopez-Barneo, 2020). The CB, classically considered to be fundamentally involved in the regulation of respiration, is now viewed as a polymodal arterial chemoreceptor needed for optimal regulation of metabolism and homeostasis of the organism (Figure 1C).

## Mitochondria-To-Membrane Signaling Model of Acute Oxygen Sensing

Acute responsiveness to hypoxia is an intrinsic property of glomus cells that is maintained in *in vitro* preparations such as dispersed cells, CB slices, or glomus cell-petrosal neuron synapse (Lopez-Barneo et al., 1988; Peers, 1990; Buckler and Vaughan-Jones, 1994; Zhong et al., 1997; Pardal et al., 2000). The curves relating cytosolic Ca^2+^ level or single glomus cell catecholamine secretion as a function of O_2_ tension (PO_2_) are remarkably similar to the hyperbolic relationship existing between afferent CB sensory activity and arterial PO_2_ (Figures 1D–F). Although the membrane events – depolarization and extracellular Ca^2+^ influx‐ underlying glomus cell responsiveness to hypoxia (known as the “membrane hypothesis”) are broadly accepted, mitochondria have also been classically considered to be involved in CB O_2_ sensing. A “metabolic hypothesis” was supported by the high sensitivity of CB to mitochondrial poisoning and the fact that mitochondrial inhibitors are powerful CB stimulants. Indeed, the existence in the CB of a special cytochrome c oxidase with low O_2_ affinity was proposed several decades ago, although it was placed in type II rather than in type I cells (Mills and Jobsis, 1972). This idea of a mitochondrial O_2_ sensor was further suggested by the analysis of light-dependent interaction of CO with heme proteins in CB cells, although as the experiments were performed in whole CB preparations, the precise cellular location of the sensor was not determined precisely (Wilson et al., 1994). In addition, Duchen and Biscoe (1992a,b) showed that in dispersed CB glomus cells mitochondrial parameters (e.g., NADH level or mitochondrial membrane potential) are highly sensitive to changes in ambient PO_2_, thereby strongly supporting the “metabolic hypothesis.” However, these last authors proposed that Ca^2+^ release from mitochondria was the signal to trigger hypoxia-induced transmitter release, a conclusion that was in direct opposition to the well-established dependence of hypoxic glomus cell activation on extracellular Ca^2+^ influx (Lopez-Barneo et al., 1993; Buckler and Vaughan-Jones, 1994; Urena et al., 1994).

### Acute O_2_ Sensing Depends on Mitochondrial Complex I Signaling

The first step to resolve to conflict between the “membrane” and “metabolic” hypotheses came from experiments on PC12 cells (an O_2_-sensitive catecholaminergic cell line; Taylor et al., 2000) and carotid body slices (Ortega-Saenz et al., 2003) showing that, as it occurs with hypoxia, catecholamine secretion induced by mitochondrial electron transport chain (ETC) inhibitors acting on complexes I, II, III, and IV is fully abolished by removal of extracellular Ca^2+^ or administration of 0.2 mM Cd^2+^, a non-selective voltage-gated Ca^2+^ channel blocker (Urena et al., 1994). Separate experiments showed that ETC blockers also inhibit the O_2_-sensitive background K^+^ current in dispersed glomus cells (Wyatt and Buckler, 2004). These data on single cells are in good agreement by previous work on whole CBs showing that dopamine secretion during incubation with cyanide is strongly inhibited in the absence of extracellular Ca^2+^ (Obeso et al., 1989). Ortega-Saenz et al. (2003) found that rotenone, a highly selective mitochondria complex I (MCI) blocker that binds near the last Fe/S cluster (N2 site) and prevents the transfer of electrons to ubiquinone, was very effective in occluding any effect of hypoxia. In contrast, activation of glomus cells by hypoglycemia was unaffected by rotenone (Garcia-Fernandez et al., 2007). These data suggested that hypoxia and hypoglycemia are sensed by separate mechanisms and that a rotenone binding site is directly involved in acute O_2_ sensing by glomus cells.

**Figure 2:**
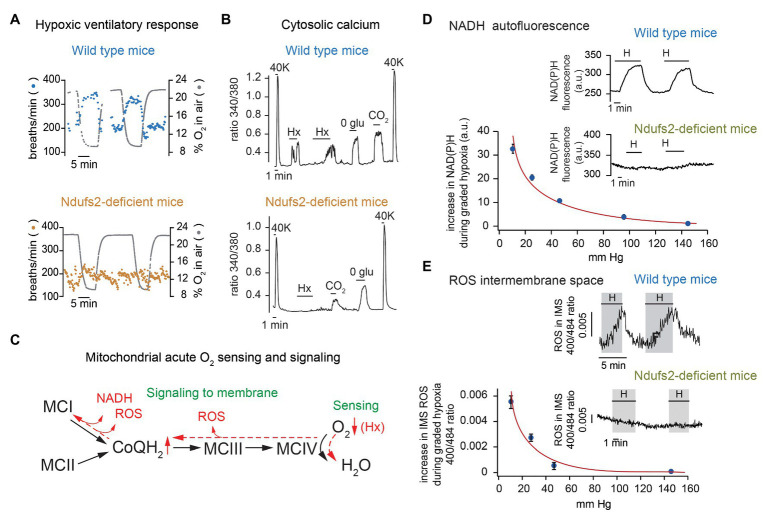
Selective inhibition of acute oxygen sensing by arterial chemoreceptors in mitochondrial complex I (MCI)-deficient mice. **(A)** Ventilatory response to hypoxia in wild type (top) and Ndufs2-deficient (bottom) mice. **(B)** Changes in cytosolic Ca^2+^ in single wild type (top) and Ndufs2-deficient (bottom) glomus cells induced by depolarization (40 mM K^+^), hypoxia (Hx, ~15 mm Hg), 0 glucose (0 glu), and hypercapnia (switching from 5 to 10% CO_2_). **(C)** Scheme of the electron transport chain illustrating the mitochondria-to-membrane signaling model of acute O_2_ sensing by glomus cells. Changes in chemical equilibrium induced by hypoxia (Hx) are represented in red. **(D)** Changes in NADH autofluorescence in single glomus cells from wild type and Ndufs2-deficient mice during exposure to hypoxia. Relationship between NADH levels and extracellular oxygen tension. **(E)** Measurement of reactive oxygen species (ROS) at the mitochondrial intermembrane space in single glomus cells from wild type and Ndufs2-deficient mice. Relationship between ROS levels and extracellular oxygen tension. Modified from Fernandez-Aguera et al. (2015) and Arias-Mayenco et al. (2018).

To investigate the role of MCI in acute O_2_ sensing, we generated conditional knockout mice lacking the *Ndufs2* gene, which codes a 49 kDa protein that contributes to the ubiquinone/rotenone binding site and is also essential for the assembly of the catalytic core in MCI (Kashani-Poor et al., 2001; Carroll et al., 2013). Because generalized bi-allelic deletion of *Ndufs2* results in embryonic lethality, we generated conditional *Ndufs2* knockout mice with either ablation of *Ndufs2* in glomus cells and other catecholaminergic cells (TH-NDUFS2 mice) or ubiquitous tamoxifen (TMX)-induced *Ndufs2* deletion in adulthood (ESR-NDUFS2 mice). TH-NDUFS2 mice had a normal development although they had smaller size than adult wild type littermates probably due to dwarfing secondary to the loss of hypothalamic dopaminergic neurons (Diaz-Castro et al., 2012; Fernandez-Aguera et al., 2015). At 2 months of age, these mice exhibited a loss of the hypoxic ventilatory response (HVR; Figure 2A), although they had a normal ventilatory response to hypercapnia (Fernandez-Aguera et al., 2015). CBs from TH-NDUFS2 mice appeared slightly hypertrophied and with normal structural organization. However, Ndufs2-deficient glomus cells showed an almost complete abolition of responsiveness to hypoxia (monitored by either the catecholamine secretory response or the changes in cytosolic [Ca^2+^]), while they responded normally to hypercapnia and hypoglycemia (Figure 2B). Similar results were observed in ESR-NDUFS2 mice, in which Ndufs2 deficiency was induced by TMX treatment in adulthood and exhibited an almost complete disappearance of MCI structure and function (Fernandez-Aguera et al., 2015; Arias-Mayenco et al., 2018). In contrast with the effects of Ndufs2 deficiency, ablation of the *Ndufs4* gene, which codes a non-essential MCI auxiliary subunit that reduces MCI activity by approximately 50% (Kruse et al., 2008), did not cause appreciable changes in the catecholamine release and cytosolic Ca^2+^ responses to hypoxia in glomus cells (Fernandez-Aguera et al., 2015). These data indicated that MCI function is essential for acute O_2_ sensing and confirmed that hypoxia and hypoglycemia are sensed by means of separate mechanisms. Interestingly, it was found that CB cells contain high levels of succinate, suggesting a highly active Kreb’s cycle, and that upregulation or downregulation of succinate dehydrogenase activity enhances or diminishes, respectively, sensitivity to hypoxia in glomus cells (Fernandez-Aguera et al., 2015; Gao et al., 2017; Arias-Mayenco et al., 2018). Although glomus cells survived several months in the absence of MCI, they rapidly died after ablation of MCII (Diaz-Castro et al., 2012; Platero-Luengo et al., 2014).

Taken together, these experimental findings suggested a model of acute O_2_ sensing in which mitochondria, acting as a sensor and effector of the hypoxic response, modulate membrane excitability. We proposed that decreased cytochrome c oxidase activity under hypoxia causes a backlog of electrons along the ETC and an increase in the ratio of reduced/oxidized ubiquinone (QH_2_/Q), which results in slowing down or even reversion of MCI with NADH accumulation and reactive oxygen species (ROS) production (Figure 2C). NADH and ROS are the signals that modulate plasmalemmal ion channels to produce depolarization and activation of glomus cells. Graded accumulation of NADH in glomus cells induced by lowering PO_2_ was abolished in Ndufs2-deficient mice (Fernandez-Aguera et al., 2015; Arias-Mayenco et al., 2018; Figure 2D). We were also able to monitor in real time the changes in mitochondrial ROS production by means of a fluorescent genetic probe targeted to either mitochondrial intermembrane space (IMS) or matrix. Using this methodology, we demonstrated that acute hypoxia induces in glomus cells a dose-dependent increase in IMS (and cytosol) ROS, which is markedly decreased by rotenone and in Ndufs2-deficient mice (Figure 2E). However, the possibility that IMS ROS produced in other sites along a reduced ETC (e.g., in MCIII) also contribute to the hypoxic response cannot be discarded (Figure 2C; Waypa et al., 2010). In support of the MMS model, we showed that intracellular dialysis of glomus cells with NADH and H_2_O_2_ mimic hypoxia (increase in input resistance and decrease in voltage-gated K^+^ current amplitude) and prevents further modulation of K^+^ channels by lowering PO_2_ (Fernandez-Aguera et al., 2015). Other mitochondrial signals (e.g., decrease in cytosolic ATP level restricted to O_2_-sensing microdomains; see below) could also contribute to modulation of membrane channels and the hypoxic response (Varas et al., 2007).

### Signature Gene Expression Profile in O_2_-Sensing Chemoreceptor Cells

In the past decades, several groups have reported gene expression data focusing on different aspects of CB glomus cell function and, recently, two such studies provided relevant clues for advancing the understanding of glomus cell acute O_2_ sensing. In one case, single neonatal glomus cell RNA sequencing confirmed the constitutive high expression of Hif2α and highlighted the elevated expression of two atypical mitochondrial subunits (Ndufa4l2 and Cox4i2), and several ion channels, in particular, Task1 and the low-threshold Ca^2+^ channel α1H subunit (Zhou et al., 2016). This work also showed the high level of expression of genes coding for molecules involved in G-protein signaling, an observation compatible with the elevated number of metabotropic ligands and receptors in glomus cells. A parallel microarray study performed in our laboratory focused on the comparative expression profile of adult CB, adrenal medulla (AM), and superior cervical ganglion (SCG), which are tissues of the same neural crest embryological origin but variable O_2_ sensitivity (CB>AM>SCG). Our work confirmed most of the genes reported in the single-cell sequencing study mentioned above and demonstrated a set of genes highly expressed in CB, and less markedly in the AM, in comparison with the SCG with a potential role in acute O_2_ sensing (Gao et al., 2017). The most relevant genes in the CB signature gene expression profile code Hif2α, three atypical mitochondrial subunits (Ndufa4l2, Cox4i2, and Cox8b), pyruvate carboxylase (Pcx), and some types of ion channels (Task1, Task3, and the α1H Ca^2+^ channel subunit). In the context of the MMS model, it was of special relevance the identification of Pcx and the three nuclear-encoded atypical mitochondrial subunits (Ndufa4l2, Cox4i2, and Cox8b), which could be responsible for the special O_2_-sensitivity of glomus cells. In particular, the high level of *Pcx* mRNA expression is compatible with the accumulation of biotin, a cofactor necessary for the function of Pcx and other carboxylases, accumulated in large quantity in glomus cells (Ortega-Saenz et al., 2016). Pcx is an anaplerotic enzyme that catalyzes the formation of oxaloacetate, thereby replenishing the pool of Krebs’s cycle intermediates required for an accelerated synthesis of substrates (NADH and FADH_2_) for the ETC. Therefore, Pcx probably contributes to the active oxidative metabolism and high O_2_ consumption characteristic of CB cells. This idea is also compatible with the high levels of succinate found in the CB and the strict dependence of CB survival and function on succinate dehydrogenase activity (Diaz-Castro et al., 2012; Platero-Luengo et al., 2014; Fernandez-Aguera et al., 2015).

### Acute O_2_ Sensing Through Hif2α-Dependent Expression of Atypical Mitochondrial Complex IV Subunits

**Figure 3:**
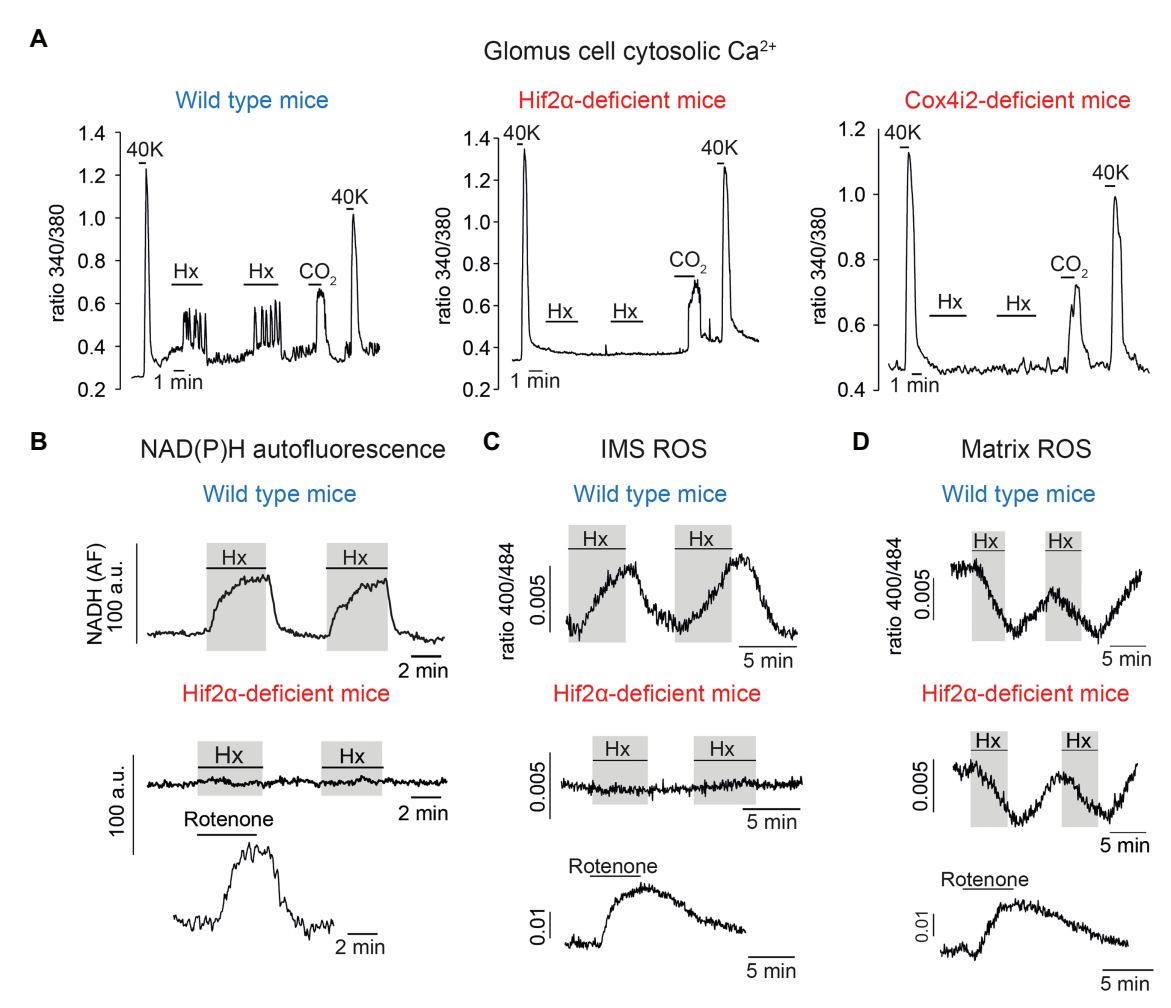
Selective inhibition of carotid body glomus cell responsiveness to hypoxia in Hif2α‐ and Cox4i2-deficient mice. **(A)** Changes in cytosolic Ca^2+^ in single wild type (left), Hif2α-deficient (center), and Cox4i2-deficient (right) glomus cells induced by depolarization (40 mM K^+^), hypoxia (Hx, ~15 mm Hg), and hypercapnia (switching from 5 to 10% CO_2_). **(B)** Changes in NADH autofluorescence in single glomus cells from wild type and Hif2α-deficient mice during exposure to hypoxia. **(C)** Measurement of ROS at the mitochondrial intermembrane space (IMS) in single glomus cells from wild type and Hif2α-deficient mice. **(D)** Measurement of ROS at the mitochondrial matrix in single glomus cells from wild type and Hif2α-deficient mice. In B-D, response to rotenone (0.5–1 μM) was tested to show the normal function of MCI. Modified from Moreno-Dominguez et al. (2020).

Although it was known long ago that Hif2α is constitutively expressed at high levels in normoxic catecholaminergic tissues (Tian et al., 1998), the role of this factor in CB function has not been studied until the last few years. It has been shown that transgenic overexpression of *Epas1* (the gene coding Hif2α) produces CB hypertrophy (Macias et al., 2014) and embryonic ablation of *Epas1* results in CB atrophy (Macias et al., 2018), thereby suggesting that Hif2α is essential for CB development and function. Heterozygous (*Epas1*+/−) mice were reported to have an exaggerated CB responsiveness to hypoxia (Peng et al., 2011) but more recent experiments performed independently by two different groups have shown that these mice have a decrease in the HVR (Hodson et al., 2016; Moreno-Dominguez et al., 2020). Inhibition of the HVR is also seen in variable degrees in mice with homozygous partial (Hodson et al., 2016) or complete (Moreno-Dominguez et al., 2020) conditional deletion of *Epas1* in adulthood. In agreement with these observations, glomus cells from conditional *Epas1*-null mice show selective abolition of the rise in cytosolic [Ca^2+^] (Figure 3A, left and center) or the secretory response to hypoxia. Moreover, NADH and IMS ROS signals induced by hypoxia are strongly inhibited in *Epas1*-deficient glomus cells (Figures 3B,C). Interestingly, the hypoxia-induced decrease in matrix ROS (Arias-Mayenco et al., 2018) was not altered by *Epas1* deficiency (Figure 3D) thereby indicating that the lack of Hif2α did not change the basic mitochondria metabolism but selectively inhibited signaling in response to low PO_2_. In parallel with these functional data, it has been shown that abolition of *Epas1* results in a selective downregulation of mRNAs coding Pcx and the atypical mitochondrial ETC subunits characteristic of CB cells (Moreno-Dominguez et al., 2020). These results are compatible with previous studies reporting that hypoxia induces Cox4i2 (Fukuda et al., 2007) and Ndufa4l2 (Tello et al., 2011) in a Hif-dependent manner (see also Aras et al., 2013), and that Cox8b promoter contains Hif binding sites (Gao et al., 2017). Together these findings indicate that the expression of Hif2α-dependent genes confer acute O_2_ responsiveness to CB glomus cells. Indeed, the *Epas1*-null phenotype (inhibition of HVR and lack of glomus cells sensitivity to hypoxia) is also observed in mice with ablation of the *Cox4i2* gene in catecholaminergic cells (TH-COX4I2 mice; Figure 3A, right; Moreno-Dominguez et al., 2020).

**Figure 4:**
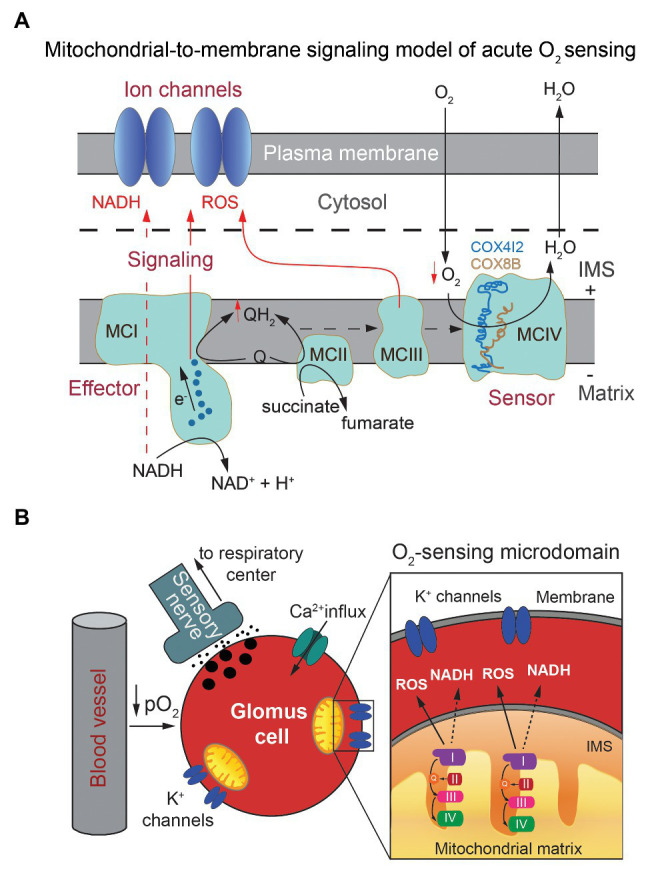
Mitochondria-to-membrane signaling model of acute oxygen sensing by glomus cells. **(A)** Scheme illustrating the mitochondrial signals (NADH and ROS) generated upon exposure to hypoxia and their interaction with membrane ion channels. Modified from Moreno-Dominguez et al. (2020). **(B)** Model of chemosensory transduction by O_2_-sensing glomus cells in the carotid body. Modified from Fernandez-Aguera et al. (2015).

The data reported so far provide molecular and mechanistic explanation for an MMS model of acute O_2_ sensing by arterial chemoreceptor cells in which cytochrome c oxidase acts as an O_2_ sensor that, depending on O_2_ availability, determines the redox state of the steps upstream in the ETC. In response to hypoxia, the increase in the reduced state of MCIII and accumulation of QH_2_ results in the generation of the signals (NADH and ROS) that modulate membrane ion channels (Figures 4A,B, see also Figure 2C). However, the precise role of each of the atypical MCIV subunits and how they influence acute O_2_ sensing in the CB and, possibly, other acutely responding organs, remains to be studied. Ndufa4l2, which is coded by one of the most abundant mRNA species in CB glomus cells, is an isoform of the most widely expressed Ndufa4 subunit, which appears to be associated to MCIV rather than to MCI (Carroll et al., 2006; Balsa et al., 2012). Ndufa4l2 is highly expressed in lung and brain pericytes and some tumor cells (Lucarelli et al., 2018) but its function is poorly known. Expression of Ndufa4l2 attenuates oxygen consumption and decreases ROS production in mitochondria (Tello et al., 2011; Meng et al., 2019), however ablation of the *Ndufa4l2* gene did not produce any clear effect on glomus cell function or HVR (Moreno-Dominguez et al., 2020). Therefore, the precise role of Ndufa4l2 in the context of acute O_2_ sensing remains to be determined. On the other hand, Cox4i2 and Cox8b are atypical isoforms of the more broadly distributed Cox4i1 and Cox8a subunits, which are part of the catalytic core of MCIV (Tsukihara et al., 1996). Besides in the CB, Cox4i2 is highly expressed in the lung and some cell types (e.g., pericytes; Huttemann et al., 2012) and Cox8b appears associated to the browning of adipose tissue (Wang et al., 2016). Interestingly, Cox4 and Cox8 subunits contain single adjacent transmembrane helices running in parallel at the periphery of MCIV (Tsukihara et al., 1996; Kadenbach and Huttemann, 2015) that, although located relatively far from the catalytic site (heme a3/CuB), could induce subtle structural changes in MCIV or in its association with supercomplexes that influence the affinity for or reaction rate with O_2_. Structural studies have suggested that the Cox8 subunit contributes to the formation of mitochondrial supercomplexes (Wu et al., 2016; Rieger et al., 2017) and a recent study on tumor cells lines have reported that expression of Cox4i2 (instead Cox4i1) decreases the Km of cytochrome c oxidase for O_2_ (Pajuelo Reguera et al., 2020). In this last study, the Km of cytochrome c oxidase for O_2_ varied between ~0.5 mm Hg (in mitochondria expressing Cox4i1) and ~1 mm Hg (in mitochondria expressing Cox4i2). These are PO_2_ values much lower than those necessary for activation of glomus cells, even assuming a steep O_2_ gradient between the extracellular medium and mitochondria. Therefore, it seems that in addition to the Cox4 subunit isoforms, other factors may influence the Km of cytochrome c oxidase for O_2_ in glomus cells. In sum, the MMS model provides a satisfactory molecular explanation for acute O_2_ sensing by arterial chemoreceptor cells, which depends on a Hif2α-dependent expression of specific genes. The special O_2_ sensitivity of glomus cells seems to result from the combination of an accelerated ETC and O_2_ consumption due to an active Krebs cycle and a relatively low affinity of cytochrome c oxidase for O_2_. In this way, electron flux in mitochondrial ETC is modulated in a physiological range of O_2_ tensions. Whether an MMS model, involving similar genes and regulatory mechanisms, also participates in acute O_2_ sensing by other tissues remains to be studied. In this regard, it is important to note that Cox4i2-deficient mice exhibit strong inhibition of hypoxic pulmonary vasoconstriction (Sommer et al., 2017), an acute response to hypoxia that, similar to hypoxic glomus cell activation, depends on the production of ROS by mitochondria and the modulation of O_2_-sensitive K^+^ channels (Weir et al., 2005).

## Clinical and Pharmacological Implications

In recent years, the CB has gained renewed medical interest due to its involvement in the pathogenesis of several highly prevalent human diseases, such as neurogenic hypertension, obstructive sleep apnea, and chronic cardiac failure. In addition, CB dysfunction also contributes to the pathophysiology of respiratory depression, a frequent complication of anesthesia and drug abuse.

### Carotid Body Inhibition

CB activation is the first line of defense against hypoxic challenges and, therefore, CB dysfunction may have fatal consequences. Indeed, bilateral resection of the CB, most commonly due neck tumor surgery or asthma treatment, leaves the patients unaware of hypoxemia (Timmers et al., 2003). These patients cannot adapt to hypoxic environments and although they appear to live unaffected in normoxic conditions, disturbances during sleep and unexplained cases of death have been reported. Genetic/developmental CB defects, such as congenital central hypoventilation syndrome (CCHS) and sudden infant death syndrome (SIDS) are life-threatening disorders partially related to alterations in CB function that, although rare in humans, can seriously impair O_2_-dependent respiratory control. CCHS is frequently associated with mutations in genes (such as *RET* or *PHOX2B*), which are relevant to development of neural crest-derived tissues (Amiel et al., 2003; Gaultier et al., 2004). Interestingly, CB glomus cells express high levels of GDNF (Villadiego et al., 2005), a neuroprotective dopaminotrophic factor that can activate RET, and genetic ablation of GDNF in adulthood results in a marked reduction in the number of TH-positive cells in the CB (Pascual et al., 2008). Decrease in size with reduction in the number of TH-positive cells and increased number of type II cells has been reported in CBs of autopsied CCHS (Cutz et al., 1997) and SIDS (Porzionato et al., 2013) patients. Prematurity and environmental factors, such as hyperoxia, retard maturation of CB chemoreceptors. Maternal smoking inhibits CB development and the excitability of AM chromaffin cells (Buttigieg et al., 2009).

The most frequent cause of CB inhibition is the use (or abuse) of anesthetics, myorelaxants, and analgesics. Volatile anesthetics (halothane and others) depress glomus cell excitability because they increase the open probability of background TASK1-like K^+^ channels (Buckler et al., 2000). Most of the myorelaxant drugs used in anesthesia are cholinergic antagonists, which interfere with the activation of the CB chemosensory synapse and inhibit the hypoxic ventilatory response (Jonsson et al., 2004). Endogenous opioids (enkephalins) are produced in the CB, where they have an auto‐ or paracrine inhibitory effect (Kirby and McQueen, 1986). Systemic administration of opioids produces a strong respiratory depression, due in part to inhibition of peripheral chemoreceptors (Pokorski and Lahiri, 1981). However, opioid-induced respiratory depression (OIRD) in conscious rats is enhanced after bilateral CB denervation, suggesting a protective rather than causative role of the CB in OIRD (Baby et al., 2018). The design of well-tolerated drugs to activate peripheral chemoreceptors, which in turn stimulate the respiratory center, is a promising strategy to alleviate OIRD in humans; a clinical condition that has become a major health problem, particularly in the United States with a toll of over 150 deaths daily. In this regard, blockers of several types of K^+^ channels are already being tested in the clinical setting as respiratory stimulants (Chokshi et al., 2015; Roozekrans et al., 2015). Within the context of this discussion, it is worth mentioning that these CB stimulants, which act downstream of the O_2_-sensing mechanism, might be useful to treat “silent hypoxemia,” a bewildering frequent clinical manifestation found in patients with coronavirus disease 19 (COVID-19), who exhibit severe hypoxemia without clear signs of distress (dyspnea) or significant acceleration of breathing (Tobin et al., 2020). Given that in the early stages of coronavirus infection human cells undergo profound changes in the expression of mitochondrial proteins (Gordon et al., 2020), a plausible explanation for “silent hypoxemia” is the alteration of the mitochondria-based O_2_-sensor in coronavirus-infected CB glomus cells (Archer et al., 2020; Tobin et al., 2020). Another aspect of the MMS model of acute O_2_ sensing with translational relevance is the identification of the mitochondrial ETC as a potential pharmacological target to stimulate respiration. In this regard, it should be tested whether MCI inhibitors, such as metformin, one of the most broadly used drugs to treat type II diabetes (Protti, 2018), can activate the CB and stimulate respiration. It could be optimal to combine metformin with novel highly membrane permeant precursors of succinate (bis-1-acetoxy-ethyl succinate or diacetoxy-methyl succinate; Ehinger et al., 2016). We have shown that increased levels of ubiquinol (CoQH_2_) resulting from the application of membrane permeant dimethyl succinate increases responsiveness to hypoxia (Arias-Mayenco et al., 2018). A combination of both therapies (metformin plus succinate prodrugs) would potentiate CB activation and at the same time prevent lactic acidosis secondary to metformin seen in some patients (Protti, 2018).

### Carotid Body Over-Activation

Chronic activation of the CB, as it occurs in patients with sleep apnea, metabolic syndrome or chronic left cardiac failure, due to intermittent hypoxia, high fat diet, or carotid hypoperfusion, respectively, can lead to exaggerated sympathetic outflow and autonomic dysfunction (see for review Ortega-Saenz and Lopez-Barneo, 2020). Although the pathophysiology of these maladaptive processes is still poorly known (Marcus et al., 2010; Paton et al., 2013; Schultz et al., 2013; Ribeiro et al., 2018), it has been shown in animal models that CB resection or deafferentation restores the sympathetic tone and improves the associated cardiovascular and metabolic alterations (Del Rio et al., 2013, 2016; McBryde et al., 2013; Ribeiro et al., 2013). However, translation of this therapy to the clinical setting has numerous limitations because the lack of CB may be cause of cardiovascular events, particularly during episodes of hypoxia and hypercapnia. CB resection could also have severe side effects such as altered glucose regulation or a reduced ability to acclimatize to high altitudes (Johnson and Joyner, 2013; Pijacka et al., 2018). In a pilot clinical trial performed on patients with chronic heart failure, bilateral CB ablation improved the autonomic imbalance but also increased the occurrence of nocturnal hypoxia, particularly in subjects with concomitant sleep apnea (Niewinski et al., 2017). An alternative to CB resection is the development of pharmacological drugs to selectively modulate CB chemosensory activity and plasticity. In this regard, it has been shown that a purinergic P2X3 receptor blocker (AF-219) inhibits CB afferent activity and alleviates hypertension in a rat model (Pijacka et al., 2016). The translation of these findings to the clinical setting may be facilitated by the fact that purinergic P2X3 receptor blockers (i.e., AF-219; also known as MK-7264 or Gefapixant) are already used in clinical trials to treat refractory chronic cough in humans, notwithstanding the unwanted side effect that taste sensation is also affected (Smith et al., 2020). A novel potential therapeutic option is represented by Hif2 antagonists, drugs already in clinical trials for the treatment of some types of cancer (Courtney et al., 2018; Fallah and Rini, 2019). Acute O_2_ sensing by glomus cells depends on Hif2α (Moreno-Dominguez et al., 2020) and systemic administration of a Hif2 inhibitor (PT2385) results in inhibition of the HVR (Cheng et al., 2020). Moreover, Hif2α is necessary for the proliferation of CB cells in hypoxia (Hodson et al., 2016) and activation of glomus cells is necessary for the proliferation and differentiation of CB progenitors and neuroblasts into mature O_2_-sensitive glomus cells (Platero-Luengo et al., 2014; Sobrino et al., 2018). Hence, Hif2 inhibitors may be beneficial to selectively modulate CB responsiveness to hypoxia and sympathetic over-activation.

### Carotid Body Tumorigenesis

Chemodectomas are rare and mostly benign CB tumors that have attracted special attention because they are often used as a model to investigate the pathogenesis of paragangliomas (PGL), tumors generated in tissues of the peripheral nervous system derived from neural crest precursors. The most common cause of hereditary CB PGL is germ line mutations in genes coding subunits of mitochondrial succinate dehydrogenase (most frequently mutations in Sdhd and Sdhc; Baysal, 2008; Her and Maher, 2015). Patients are heterozygous (with a normal and a mutated allele) and tumorigenesis is believed to be triggered by the loss of the normal allele (loss of heterozygosity) in CB glomus cells. However, the reasons why this allele is lost in some cell types (e.g., cells in CB and other paraganglia) and not in others as well as the mechanisms leading to tumor formation are unknown (Millan-Ucles et al., 2014). Given that the histology of CB PGL resembles that of hypertrophied CBs seen in chronically hypoxic subjects and that PGL incidence increases in populations living at high altitude (Astrom et al., 2003), a widely accepted hypothesis of tumor generation is the so called “pseudo hypoxic drive” (Selak et al., 2005; Smith et al., 2007). This hypothesis is based on the fact that succinate accumulation, secondary to succinate dehydrogenase dysfunction, causes downstream inhibition of prolyl hydroxylases involved in normal degradation of Hif as well as inhibition of histone demethylases and other enzymes, thereby causing cell proliferation. Indeed, overexpression of nondegradable Hif2α (but not Hif1α) induces CB hypertrophy (Macias et al., 2014). Moreover, deletion of the gene coding for prolyl hydroxylase 2 in mice induces Hif2α-dependent CB glomus cell proliferation with a PGL-like phenotype (Fielding et al., 2018). However, experimental evidence indicates that unlike humans, heterozygosity for mutations in succinate dehydrogenase subunits does not predispose mice to PGL. Adult knockout mice heterozygous for *Sdhd* show practically normal CB function, with only a subtle glomus cell hyperplasia and organ hypertrophy (Piruat et al., 2004). In addition, conditional (embryonic or adult) bi-allelic ablation of *Sdhd* causes a marked glomus cell loss (Diaz-Castro et al., 2012). It seems therefore that in addition to succinate dehydrogenase subunit mutations, other hits, related to animal species, age, or cell metabolism, are necessary for tumorigenesis *in vivo*. Although there is convincing *in vitro* and *in vivo* evidence that multipotent stem cells contribute to CB angiogenesis and expansion of parenchyma during exposure to sustained hypoxia (Pardal et al., 2007; Annese et al., 2017), it has also been shown that proliferation of TH-positive cells greatly contributes to the growth of the glomus cell pool during the first 2–3 days of hypoxia (Paciga et al., 1999; Chen et al., 2007; Wang et al., 2008; Fielding et al., 2018). In the rat, and probably also in other species, this initial glomus cell expansion is due to proliferation and maturation of a population of TH-positive neuroblasts, which differentiate into O_2_-sensing glomus cells (Sobrino et al., 2018). Because hypoxia does not seem to induce proliferation of CB stem cells and undifferentiated progenitors *in vitro* (Platero-Luengo et al., 2014), a fundamental question that remains to be answered is whether hypoxia-induced release of transmitter and cytokines by mature glomus cells is a critical paracrine signal to trigger CB TH-positive cell proliferation and possibly the initial stages of tumor transformation. This would explain why Hif2α stabilization increases CB growth and the expansion of TH-positive cell population, and it would also support the use of Hif2 antagonists as potential therapeutic options to prevent CB PGL formation and growth.

## Conclusions and Future Directions

The knowledge of CB physiology and the sensory function of glomus cells have steadily advanced in the last years. In addition to their well-established role as arterial O_2_/CO_2_ sensors, with a major impact on the regulation of respiration, glomus cells are now considered polymodal receptors with a wide physiological impact and able to detect and integrate changes in numerous chemical and physical variables in the blood. Although the molecular mechanisms underlying glomus cell acute responsiveness to hypoxia have remained elusive, the MMS model summarized in this paper has provided an unprecedented integrated view of glomus cell function that robustly explains most of the data available and, in addition, can be further tested experimentally. The progress in the understanding of the molecular physiology of acute O_2_ sensing by glomus cells, the prototypical O_2_ sensors, will surely boost advances in the identification and characterization of other acute O_2_ sensing cells in the body and in the investigation of their pathophysiological relevance. The MMS has also unraveled novel potential targets for pharmacological modulation of CB output that could be of therapeutic applicability in highly prevalent medical disorders presenting CB dysfunction. A more complete and comprehensive view of CB physiology will surely come from studies focusing on the mechanisms of CB plasticity and their impact on the pathogenesis of human diseases. In parallel, future research should also focus on the elucidation of the molecular bases of glomus cell responsiveness to stimuli, such as changes in blood glucose, lactate or flow, as well as in circulating hormones, which are still poorly known.

## Author Contributions

PO-S and JL-B prepared the first draft of the manuscript and figures. PO-S, JL-B, AM-D, and LG contributed to the writing of the final version of the paper. All authors contributed to the article and approved the submitted version.

### Conflict of Interest

The authors declare that the research was conducted in the absence of any commercial or financial relationships that could be construed as a potential conflict of interest.
